# Tryptophan Metabolites in the Progression of Liver Diseases

**DOI:** 10.3390/biom14111449

**Published:** 2024-11-15

**Authors:** Maria Reshetova, Pavel Markin, Svetlana Appolonova, Ismail Yunusov, Oksana Zolnikova, Elena Bueverova, Natiya Dzhakhaya, Maria Zharkova, Elena Poluektova, Roman Maslennikov, Vladimir Ivashkin

**Affiliations:** 1 Department of Internal Medicine, Gastroenterology and Hepatology, I.M. Sechenov First Moscow State Medical University, 119435 Moscow, Russia; yunusov_i_r@student.sechenov.ru (I.Y.); zolnikova_o_yu@staff.sechenov.ru (O.Z.); bueverova_e_l@staff.sechenov.ru (E.B.); dzhakhaya_n_l@staff.sechenov.ru (N.D.); zharkova_m_s@staff.sechenov.ru (M.Z.); poluektova_e_a@staff.sechenov.ru (E.P.); maslennikov_r_v@staff.sechenov.ru (R.M.); ivashkin_v_t@staff.sechenov.ru (V.I.); 2 Centre of Biopharmaceutical Analysis and Metabolomics, I.M. Sechenov First Moscow State Medical University, 119435 Moscow, Russia; markin_p_a@staff.sechenov.ru (P.M.); appolonova_s_a@staff.sechenov.ru (S.A.); 3 The Interregional Public Organization “Scientific Community for the Promotion of the Clinical Study of the Human Microbiome”, 119121 Moscow, Russia

**Keywords:** MAFLD, ALD, liver cirrhosis, metabolomic profiling, tryptophan metabolite

## Abstract

The aim of this study was to investigate the levels of various tryptophan metabolites in patients with alcoholic liver disease (ALD) and metabolic-associated fatty liver disease (MAFLD) at different stages of the disease. The present study included 44 patients diagnosed with MAFLD, 40 patients diagnosed with ALD, and 14 healthy individuals in the control group. The levels of tryptophan and its 16 metabolites (3-OH anthranilic acid, 5-hydroxytryptophan, 5-methoxytryptamine, 6-hydroxymelatonin, indole-3-acetic acid, indole-3-butyric, indole-3-carboxaldehyde, indole-3-lactic acid, indole-3-propionic acid, kynurenic acid, kynurenine, melatonin, quinolinic acid, serotonin, tryptamine, and xanthurenic acid) in the serum were determined via high-performance liquid chromatography and tandem mass spectrometry. In patients with cirrhosis resulting from MAFLD and ALD, there are significant divergent changes in the serotonin and kynurenine pathways of tryptophan catabolism as the disease progresses. All patients with cirrhosis showed a decrease in serotonin levels (^MAFLD^ *p* = 0.038; ^ALD^ *p* < 0.001) and an increase in kynurenine levels (^MAFLD^ *p* = 0.032; ^ALD^ *p* = 0.010). A negative correlation has been established between serotonin levels and the FIB-4 index (*p* < 0.001). The decrease in serotonin pathway metabolites was associated with manifestations of portal hypertension (*p* = 0.026), the development of hepatocellular insufficiency (*p* = 0.008) (hypoalbuminemia; hypocoagulation), and jaundice (*p* < 0.001), while changes in the kynurenine pathway metabolite xanthurenic acid were associated with the development of hepatic encephalopathy (*p* = 0.044). Depending on the etiological factors of cirrhosis, disturbances in the metabolic profile may be involved in various pathogenetic pathways.

## 1. Introduction

Liver cirrhosis is a leading cause of mortality among patients with chronic liver disease worldwide. According to WHO data (for 2019), 2.4% of these deaths were associated with cirrhosis [1]. Moreover, mathematical forecasts suggest an increase in prevalence and mortality rates in the coming decade.

According to recent epidemiological studies, the leading etiological factors for the development of cirrhosis remain hepatitis B and C viruses, followed by alcoholic liver disease (ALD) and metabolic-associated fatty liver disease (MAFLD) [2,3]. However, given the increase in global obesity rates, insulin resistance, and alcohol consumption, it should be expected that ALD and MAFLD will soon become the dominant causes of cirrhosis [4,5].

**Figure 1:**
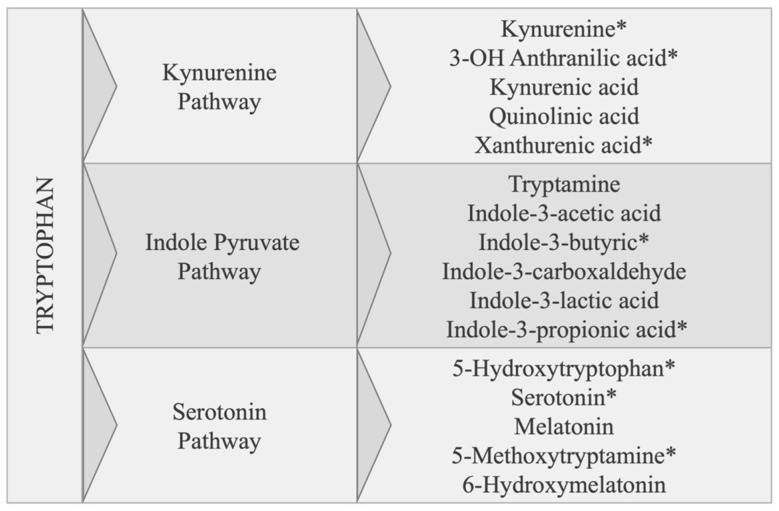
The main pathways of tryptophan catabolism in the human body. Kynurenine pathway in host immune cells and liver. Indole pyruvate pathway performed by gut microbiota. Serotonin pathway performed by enterochromaffin cells. *—metabolites whose levels were statistically highly correlated in our study with the etiology of the disease, the type of liver damage, and clinical manifestations of the disease.

This position determines the interest in conducting research aimed at developing new diagnostic methods and therapeutic strategies for ALD and MAFLD. Increasing data from recent years demonstrate the variability of human metabolomic profiling against the background of various diseases [6]. This is largely due to changes in the composition and biodiversity and the decreased metabolic activity of the microbiota. The role of the latter is actively discussed in the pathogenesis of liver diseases, including MAFLD [7,8]. It has been established that the intestinal microbiota and their metabolic activity change with the progression of liver fibrosis and are important in the development of hepatic encephalopathy in patients with decompensated liver cirrhosis [9,10,11]. Changes have been identified in the levels of short-chain fatty acids and bacterial metabolites formed during the breakdown of dietary fibers in the intestine [12]. The influence of other microbial-origin molecules, particularly tryptophan and its derivatives, is being studied. Tryptophan is an essential aromatic amino acid that enters the human body with food (fish and seafood, meat, dairy, eggs, nuts and seeds, vegetables and fruits, grains, tofu and chocolate) and is a precursor to many vital metabolites that exert various systemic effects in the body [13]. The majority of tryptophan is absorbed in the small intestine. In the peripheral bloodstream, 80–90% of tryptophan binds to albumin and subsequently enters the main metabolic pathway (Figure 1), kynurenine (90%). Tryptophan is further metabolized due to the main enzymes indoleamine-2,3-dioxygenase and tryptophan-2,3-dioxygenase. Indoleamine-2,3-dioxygenase is produced by immune cells of the peripheral blood, dendritic cells, microglia, and epithelial cells, while tryptophan-2,3-dioxygenase is predominantly expressed in the liver. A portion of the tryptophan not absorbed in the small intestine undergoes microbial catabolism in the indole pathway. A variety of symbiotic Gram-negative and Gram-positive bacteria metabolize tryptophan into indole and other derivatives using various enzymes. The involvement of Clostridium spp., Bacteroides spp., and Escherichia coli in indole synthesis using the enzyme tryptophanase has been described. Tryptophan can also be hydrolyzed by decarboxylases of Ruminococcus gnavus and Clostridium sporogenes. Clostridium sporogenes can also convert tryptophan into idole-3-pyruvic acid. Indole-3-acetic acid is synthesized, in particular, by Bacteroides, Bifidobacteria and Eubacteria. Indole-3-pyruvate is directly synthesized from tryptophan by the enzyme aromatic amino acid transaminase [14]. Enterochromaffin cells of the intestinal mucosa participate in the third metabolic pathway of tryptophan, serotonin [15].

The aim of this study was to assess the levels of various tryptophan metabolites in the serum of patients with MAFLD and ALD at different stages of the disease.

## 2. Materials and Methods

This study was conducted in accordance with the Declaration of Helsinki and approved by the ethics committee of the Sechenov First Moscow State Medical University (Sechenov University), № 01-22 dated 20 January 2022, in the period from 22 February 2022 to 14 February 2023. Patients with a confirmed diagnosis of MAFLD were included in the study. The diagnosis was established based on the patient’s history, clinical presentation, laboratory and instrumental examination data, and by excluding other etiological factors of liver damage (diagnosis per exclusionem). If alcohol consumption in hepatotoxic doses was identified for more than 6 months (>30 g/day for men and >20 g/day for women), a diagnosis of ALD was established. All patients were screened for markers of viral hepatitis (HBsAg, HBeAg, Anti-HBc-total, Anti-HBe, Anti-HBs, Anti-HCV-total, Anti-HAV-IgM, and Anti-HAV-IgG), primary biliary cholangitis and autoimmune hepatitis (AMA, ANA, SMA and anti-LKM1), as well as hemochromatosis (ferritin level, transferrin saturation with iron) and Wilson’s disease (ceruloplasmin level, Kaiser–Fleischer rings, 24 h urinary copper, and free plasma copper level). The patients also underwent an ultrasound examination of the abdominal organs and the assessment of the alpha-fetoprotein level to exclude liver and abdominal organ neoplasms. The diagnosis of steatohepatitis/cirrhosis was established based on liver biopsy data or a combination of clinical, laboratory, and instrumental data.

The control group consisted of healthy volunteers who had no gastrointestinal complaints, no comorbidities of the respiratory, urinary, endocrine, or cardiovascular system, and no oncological diseases and who visited the clinic for a preventive examination.

### 2.1. Metabolomic Analysis

At the first stage, upon the patient’s admission to the hospital, blood was drawn from the brachial vein into 2 mL vacuum tubes with the clot activator. The blood samples were centrifuged under standard conditions at room temperature at an acceleration of 3000× *g* for 10 min. After precipitation, the supernatant, serum, was poured into polypropylene Eppendorf tubes. The samples were stored at temperatures below −80 °C until the analysis stage.

At the second stage, to prepare the solution of internal standards for tryptophan metabolite analysis, samples of isotopically labeled standards sufficient for the preparation of 1 mL of solution with a concentration of 1 mg/mL in glass vials and dissolved in 1 mL of solvent were weighed. The resulting solutions were divided into 20 μL aliquots and stored at a temperature of −70 °C.

After thawing, 40 μL of the internal standard mix (IS mix) was added to 10 μL of the plasma samples in polypropylene Eppendorf tubes. The resulting mixture was evaporated to dryness in a centrifugal vacuum evaporator at a temperature of 40 °C. To the dry residue, a derivatization mixture (a solution of phenylisothiocyanate in pyridine) was added, and then the solution was mixed on a vortex for 10 s and left at room temperature for 20 min. The samples were then evaporated to dryness again in a centrifugal vacuum evaporator at a temperature of 40 °C. To the dry residue, 100 μL of a 5 mM ammonium acetate solution in methanol was added. The samples were mixed in a shaker for 30 min. To the plasma samples and laboratory plasma samples, 100 μL of deionized water was added, and to the standard solutions (Cal1-6, QCs), 100 μL of a 0.1 PBS solution was added. The resulting solutions were mixed in a vortex for 10 s and centrifuged at 14,900× *g* and a temperature of 20 °C for 10 min. A supernatant volume of 150 μL was transferred to a polypropylene microvial for subsequent chromatography–mass spectrometry analysis.

In the course of high-performance liquid chromatography and tandem mass spectrometry (HPLC-MS/MS), the identification of compounds in the sample was conducted based on two characteristics, retention time and characteristic mass spectrometric transitions. The chromatographic separation of substances was performed using gradient elution. Mass spectrometric detection was performed in the multiple reaction monitoring (MRM) mode.

Parameters of the high-performance liquid chromatography: analytical column for HPLC: ACQUITY UPLC BEH C18, 2.1 × 50 mm Particle size 1.7 μm, pre-column filter ACQUITY UPLC BEH C18. The temperature control of the analytical column was 40 °C. The flow rate of the mobile phase was 0.50 mL/min. The composition of the mobile phase: phase a—0.1% formic acid solution in deionized water; phase b—100% acetonitrile for chromatography. Sample injection volume: 5 μL. Total analysis time: 5 min.

Parameters of the tandem quadrupole mass spectrometric detector: type of ionization: electrospray with heated nebulizing gas flow (Agilent Jet Stream, USA). Gas temperature 300 °C. Gas flow 11 L/min. Nebulizer 35 psi. Sheath gas temp 300 °C. Sheath gas flow 11 L/min. Capillary voltage (positive) 3500 kV. Capillary voltage (positive) 2500 kV. Mode of analysis dynamic MRM, cycle time 600 ms.

After the analysis was completed, the obtained data were processed using the MassHunter software B.08.00 with the construction of a calibration curve. The result of the quantitative determination of the samples was outputted in the form of a general Excel file containing sample numbers and analyte concentrations.

We conducted an analysis of tryptophan and its 16 metabolites: 3-OH anthranilic acid, 5-hydroxytryptophan, 5-methoxytryptamine, 6-hydroxymelatonin, indole-3-acetic acid, indole-3-butyric, indole-3-carboxaldehyde, indole-3-lactic acid, indole-3-propionic acid, kynurenic acid, kynurenine, melatonin, quinolinic acid, serotonin, tryptamine, and xanthurenic acid. After a thorough statistical analysis, we selected only metabolites of which the levels changed significantly and correlated with the etiology of the disease or type of liver damage (Figure 1).

### 2.2. Statistical Analysis

The patient database was maintained using the MS Excel program. Statistical analysis was performed using StatTech v. 4.5.0 (Developer—StatTech LLC, Russia) and STATISTICA 12 software (StatSoft Inc., Tulsa, OK, USA). Quantitative indicators with a normal distribution were described using mean values (M) and standard deviations (SDs) at the boundaries of the 95% confidence interval (95% CI). In the absence of a normal distribution, quantitative data were described using the median (Me) and the lower and upper quartiles (Q1–Q3). For comparison of the two groups based on a quantitative indicator with a normal distribution, under the condition of the equality of variances, we used Student’s *t*-test, and for unequal variances, we used the Welch *t*-test. The comparison of two groups based on a quantitative indicator with a non-normal distribution was performed using the Mann–Whitney U-test. The comparison of three or more groups based on a quantitative indicator with a normal distribution was performed using a one-way analysis of variance, with post hoc comparisons using Tukey’s test (assuming equal variances) or the Games–Howell test (in the case of unequal variances). The comparison of three or more groups based on a quantitative indicator with a non-normal distribution was performed using the Kruskal–Wallis test, with post hoc comparisons using Dunn’s test with Holm’s correction. Multiple regression was performed with body mass index as a confounder. The direction and strength of the correlation between two quantitative indicators were assessed using Pearson’s correlation coefficient (for normally distributed indicators) or Spearman’s rank correlation coefficient (for non-normally distributed indicators). For assessing the diagnostic power of certain metabolites and of the combined tryptophan metabolic panel, receiver operating characteristic (ROC) analysis was performed using the logistic regression classification model. Differences were considered statistically significant at *p* < 0.05.

## 3. Results

The study included 84 patients—44 patients diagnosed with MAFLD, 40 patients diagnosed with ALD, and 14 healthy volunteers. The groups were comparable in terms of gender and age. The main clinical and laboratory signs of the studied groups are presented in Table S1.

Compared with the control group in Table 1, patients with MAFLD at different stages of the disease showed a statistically significant higher BMI and elevated HDL levels (*p* < 0.001). In the MAFLD group at the steatosis stage, there was a decrease in HDL and an increase in LDL and VLDL levels. The increase in VLDL was also characteristic of patients in the steatohepatitis (*p* < 0.001) and cirrhosis (*p* < 0.001) groups. Patients with cirrhosis resulting from MAFLD showed a statistically significant elevated fasting plasma glucose level (*p* = 0.026).

In the group of individuals suffering from ALD, compared to the control group, an increase in IgA levels was found at the hepatitis (*p* = 0.009) and cirrhosis (*p* < 0.001) stages, with a tendency for this indicator to increase as the disease progressed.

In both ALD and MAFLD, at the steatohepatitis stage, there was a statistically significant increase in ALT and AST levels compared to the liver steatosis group and the control group (*p* < 0.001).

In the course of the study, we analyzed the levels of tryptophan metabolites depending on the etiological factor in patients with MAFLD and ALD compared to the control group. The analysis revealed statistically significant differences between the levels of 3-OH anthranilic acid, kynurenine, 5-hydroxytryptophan, 5-methoxytryptamine, and serotonin depending on the etiological factor of liver damage (Table 2). Patients with MAFLD were distinguished from patients with ALD by elevated levels of 3-OH anthranilic acid, as well as elevated levels of serotonin and other metabolites of the serotonin pathway, 5-hydroxytryptophan and 5-methoxytryptamine. In turn, compared to the control group, there was a statistically significant decrease in serotonin levels and an increase in metabolites of the kynurenine pathway of tryptophan metabolism, such as kynurenine and 3-OH anthranilic acid (Table 2). When multiple regression was performed with body mass index as a confounder, there was no significant difference in 3-OH-anthranilic acid levels between the MAFLD and control groups (*p* = 0.085).

We also conducted an analysis of the aforementioned metabolites in patients at different stages of the disease, which also revealed changes in the levels of metabolites of the kynurenine and serotonin pathways (Table 3).

Comparison with the control group showed that serotonin and kynurenine levels demonstrated significant changes in patients with liver cirrhosis (n = 40) of various etiologies (MAFLD and ALD) (Table 3).

These data allowed us to further formulate a hypothesis about the alteration of tryptophan metabolism as the disease progresses to liver cirrhosis.

We found that as the disease progresses, there is a divergent correlation between different tryptophan metabolism pathways (serotonin and kynurenine). In patients with cirrhosis of the liver, both in the outcome of MAFLD and in the outcome of ALD, there was a statistically significant increase in kynurenine levels (*p* = 0.032, *p* = 0.010) and a statistically significant decrease in serotonin levels (*p* = 0.038, *p* < 0.001) compared to the control group. In the group of patients with alcohol-induced cirrhosis, a decrease in serotonin levels was also noted compared to steatosis (*p* = 0.048) and hepatitis (*p* = 0.026). According to the Child–Pugh scale, serotonin levels were significantly reduced in patients with class C cirrhosis compared to class A (*p* = 0.003), as were the levels of its precursor 5-hydroxytryptophan (*p* = 0.036). At the same time, in the ALD patient group, patients with steatosis (*p* = 0.048) and hepatitis (*p* = 0.026) had higher serotonin levels than patients with cirrhosis.

It is extremely important, in our opinion, to note the statistically significant negative correlation between serotonin levels and the FIB-4 index (*p* < 0.001), as well as the serotonin pathway metabolite, 5-methoxytryptamine (*p* < 0.001).

**Figure 2:**
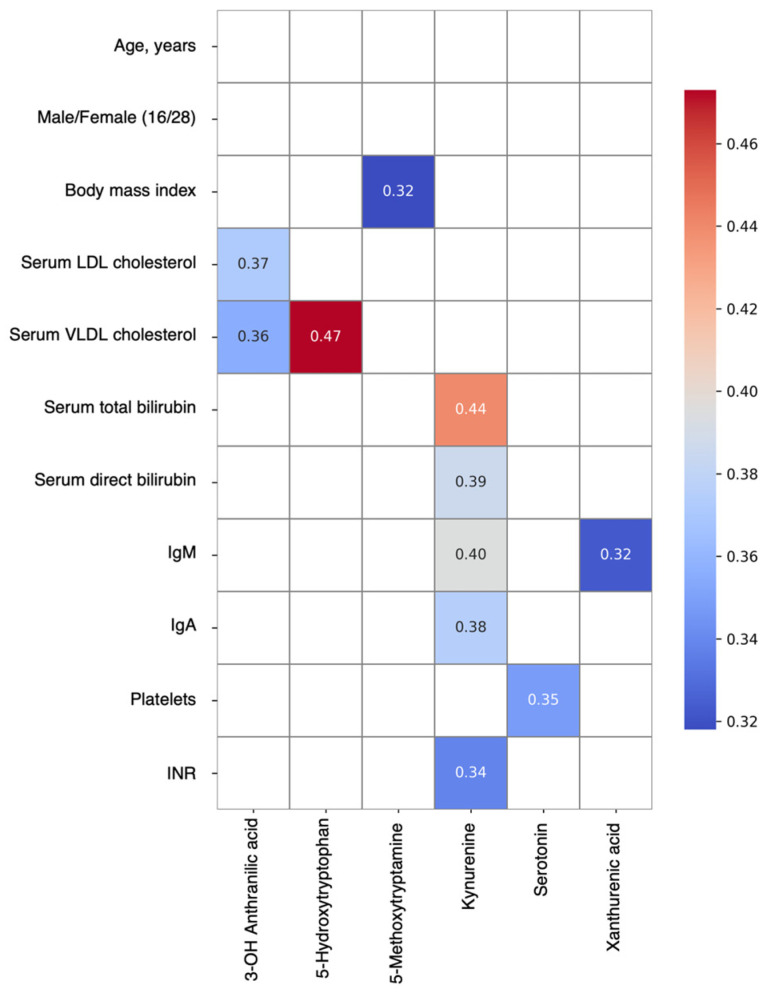
Tryptophan metabolites’ correlation analysis depending on laboratory test for MAFLD.

**Figure 3:**
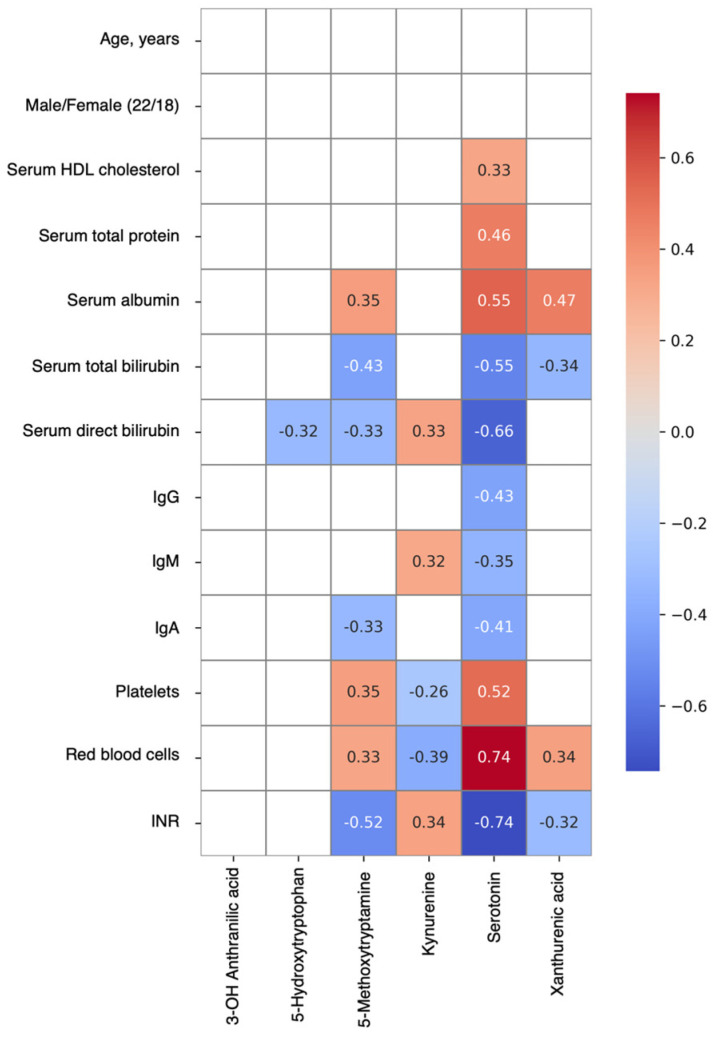
Tryptophan metabolites’ correlation analysis depending on laboratory test for ALD.

The levels of serotonin (*p* = 0.048) and 5-methoxytryptamine (*p* = 0.024) in patients with liver cirrhosis of various etiologies positively correlate with platelet levels, which are significantly lower than in the control group (*p* < 0.001) and decrease as the disease progresses to cirrhosis (Table 1, Figure 2 and Figure 3). In the analysis of the correlation between serotonin levels in patients with ALD and the parameters of the complete blood count (CBC) and coagulation profile, a significant negative correlation was noted between the levels of this metabolite (ρ = −0.743; *p* <0.001) and 5-methoxytryptamine (ρ = −0.520; *p* <0.001) and the INR indicator. Kynurenine levels positively correlate with the INR indicator in both patients with ALD (ρ = 0.340, *p* = 0.032) and those with MAFLD (ρ = 0.338; *p* = 0.025) (Figure 2 and Figure 3). In addition, a positive correlation was noted between serotonin levels and total protein (ρ = 0.463, *p* = 0.003) and albumin (ρ = 0.548, *p* < 0.001) levels, as well as HDL cholesterol levels (ρ = 0.329, *p* = 0.038), in the ALD patient group. Negative correlations were observed with total and direct bilirubin levels, as well as immunoglobulin levels (IgA (ρ = −0.41, *p* = 0.008), IgM (ρ = −0.352, *p* = 0.026), and IgG (ρ = −0.427, *p* = 0.006)). These and other statistically significant results of correlations between metabolite levels and CBC and biochemical analysis parameters are presented in Tables S2 and S3.

**Figure 4:**
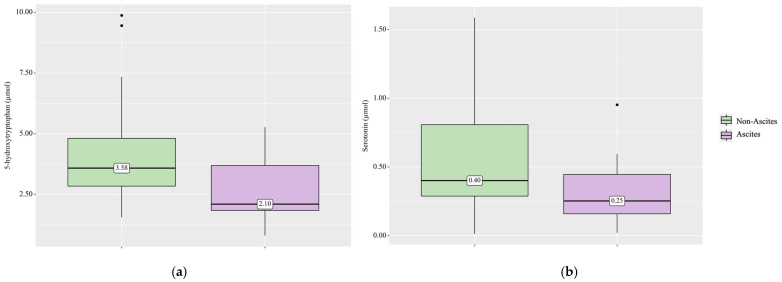
Metabolite levels in the group of patients with cirrhosis of various etiologies (MAFLD and ALD) with and without ascites. (**a**) Level of 5-hydroxytryptophan; (**b**) level of serotonin.

A detailed analysis of the liver cirrhosis group showed that the development of ascites was associated with a decrease in serotonin levels (*p* = 0.031) and its precursor 5-hydroxytryptophan (*p* = 0.013) (Figure 4).

Additionally, in patients with signs of portal hypertension based on abdominal ultrasound data (splenomegaly and/or increased diameter of portal system vessels and/or portovenous collaterals and/or ascites), there was a statistically significant decrease in serotonin (*p* = 0.026), an increase in indole pathway metabolite indole-3-butyric acid (*p* = 0.039), and a decrease in indole-3-propionic acid (*p* = 0.035).

**Figure 5:**
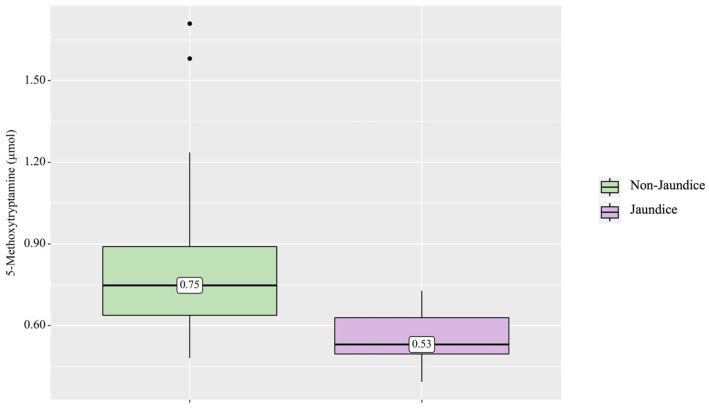
Level of 5-methoxytryptamine in the group of patients with cirrhosis of various etiologies (MAFLD and ALD) with and without the clinical syndrome of jaundice.

**Figure 6:**
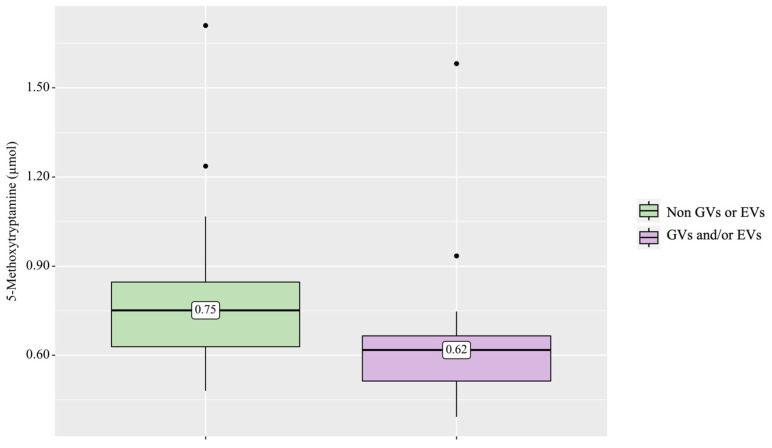
Level of 5-methoxytryptamine in the group of patients with cirrhosis of various etiologies (MAFLD and ALD) with signs of portal hypertension based on EGD data (GVs and/or EVs) and without signs of portal hypertension.

It was found that the decrease in the serotonin pathway metabolite, 5-methoxytryptamine, was associated with the development of jaundice (*p* < 0.001) in patients with cirrhosis, as well as with the presence of portal hypertension based on esophagogastroduodenoscopy (EGD) data (*p* = 0.018) (endoscopic appearance of gastric varices (GVs) and/or esophageal varices (EVs)) (Figure 5 and Figure 6).

**Figure 7:**
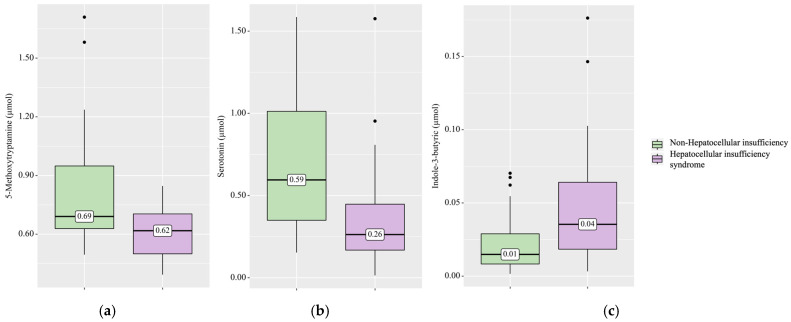
Levels of metabolites in the group of patients with cirrhosis of various etiologies (MAFLD and ALD) with and without hepatic failure syndrome (hypocoagulation, hypoalbuminemia). (**a**) Level of 5-methoxytryptamine; (**b**) level of serotonin; (**c**) level of indole-3-butyric acid.

The kynurenine pathway metabolite, xanthurenic acid, is associated with the development of hepatic encephalopathy (*p* = 0.044). An increase in the level of the metabolite of the indole pathway (indole–3-butyric acid (*p* = 0.034)) and a decrease in serotonin (*p* = 0.008) and 5-methoxytryptamine (*p* = 0.019) were found to be associated with the development of hepatocellular insufficiency (hypoalbuminemia, hypocoagulation) (Figure 7).

**Figure 8:**
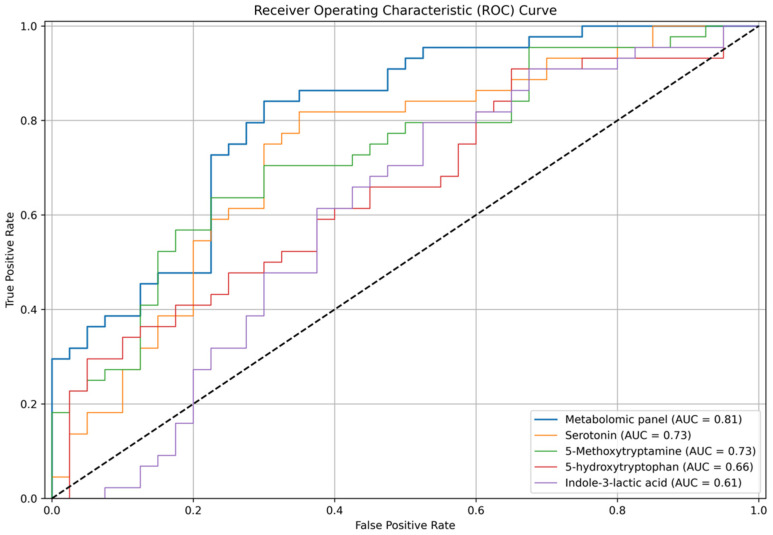
Receiver operating characteristic (ROC) analysis for and metabolic panel of 17 tryptophan metabolites (MAFLD vs. ALD).

Additionally, receiver operating characteristic (ROC) analysis was performed using the logistic regression classification model assessing the diagnostic power for certain metabolites and the combined tryptophan metabolic panel. The area under curves (AUCs) for “the best” diagnostic metabolites (serotonine, 5-methoxytryptamine, 5-hydroxytryptophan, indole-3-lactic acid) and metabolic panel are presented in Figure 8. The metabolomic panel consisting of all 17 tryptophan metabolites is more stable for determining the etiology of liver disease (MAFLD vs. ALD). The AUC for the metabolic panel is 0.81.

## 4. Discussion

**Scheme 1:**
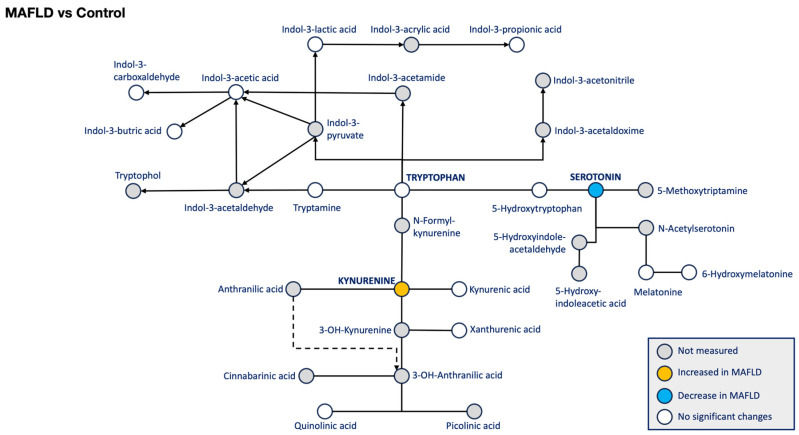
Changes in the tryptophan metabolite levels in patients with MAFLD compared to the control group.

**Scheme 2:**
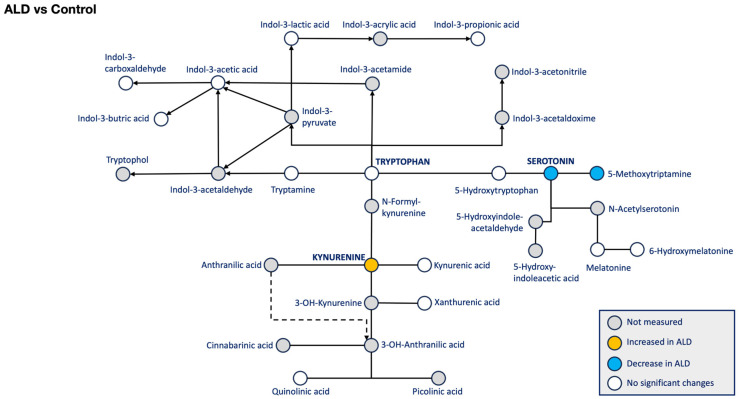
Changes in the tryptophan metabolites levels in patients with ALD compared to the control group.

**Scheme 3:**
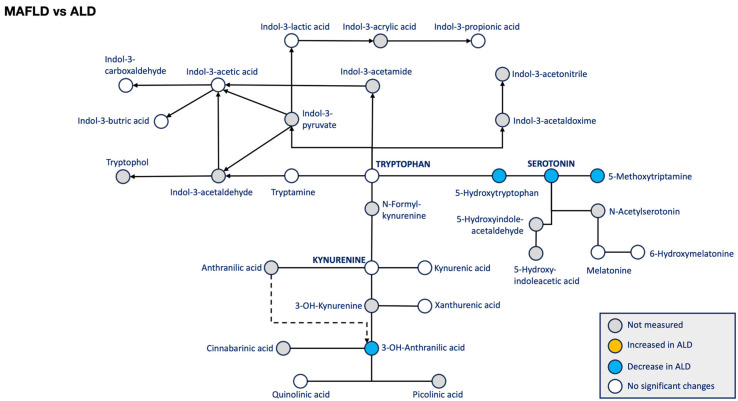
Changes in the tryptophan metabolites levels in patients with MAFLD compared to ALD.

In the course of our study, we traced changes in the levels of various tryptophan metabolites in patients with MAFLD and ALD (Scheme 1, Scheme 2 and Scheme 3). We established that as the disease progresses to cirrhosis, significant changes occur in two tryptophan catabolism pathways—the serotonin and kynurenine pathways. This results in the synthesis of serotonin and kynurenine and their derivatives.

### 4.1. Serotonin

Serotonin is a small molecule that is formed during tryptophan catabolism. Generally, 90% of serotonin is synthesized in enterochromaffin cells of the intestine under the influence of a specific enzyme, tryptophan hydroxylase 1 [16,17], with the remaining 10% synthesized in the brain. Several studies demonstrate the influence of the intestinal microbiota on serotonin metabolism in the colon. Jessica M Yano et al., in a study on germ-free mice, demonstrated a decrease in serotonin synthesis in the colon and a compensatory increase in the expression of the specific transporter 5-HT SLC6A4 [13]. Christopher S. Reigstad et al., using a mouse model, established that the expression of the enzyme tryptophan hydroxylase 1 may depend on the metabolic activity of the microbiota, particularly in relation to the production of short-chain fatty acids [18,19]. Previously, we also showed that as the disease progresses to cirrhosis, statistically significant changes are observed in the level of short-chain fatty acids in stool samples from the patients [12].

Currently, about 15 different serotonin receptors have been described [20], the most well-known of which are located in the central nervous system (CNS). However, the so-called peripheral serotonin, which is formed in the intestine, is unable to cross the blood–brain barrier and, by binding to specific receptors, functions as an endocrine factor and affects the homeostasis of the entire body [21]. After serotonin enters the systemic circulation, part of the metabolite binds to platelets, while part freely circulates in the plasma. Previous studies have shown that in addition to transporting the metabolite to target organs, platelets can express specific serotonin receptors. In the presence of other platelet adhesion activators, serotonin can potentiate the blood clotting process by binding to the 5-HT2 receptor [22,23]. In the course of our study, it was shown that low levels of serotonin and 5-methoxytryptamine negatively correlate with blood INR in patients with cirrhosis.

The role of serotonin in the pathophysiology of liver diseases has been described. A number of HT-5 serotonin receptors located on the hepatocytes, cholangiocytes and stellate cells of the liver have been studied [24]. The literature presents both positive and negative effects of serotonin on liver physiology. In a mouse model, it has been shown that serotonin participates in liver regeneration, restores tissue perfusion in the liver, and regulates microcirculation [25,26,27]. At the same time, results have been published showing that serotonin exacerbates the course of viral hepatitis and plays a crucial role in the progression of liver fibrosis [24,28,29].

We found statistically significant reductions in serotonin levels in patients with cirrhosis compared to the control group, as well as in patients with ALD, while a decrease in this metabolite was observed in comparison with the MAFLD group. In our study, low serotonin levels in patients with cirrhosis were significantly correlated with the FIB-4 index, as well as the development of ascites and hepatocellular insufficiency (hypoalbuminemia, hypocoagulation). These data are somewhat consistent with those from several other studies. In a previously published study by Beaudry P. et al., the total, intraplatelet (conjugated), and serum (non-conjugated) serotonin levels were analyzed in patients with cirrhosis (ALD and viral etiology) using a radio-enzymatic method. The authors noted a significant reduction in the total amount of serotonin in the blood of patients with cirrhosis compared to the control group; however, the level of non-conjugated serotonin was higher in the context of cirrhosis [30]. Another study on a small sample using high-performance liquid chromatography revealed a reduction in intraplatelet serotonin levels in patients with cirrhosis (ALD and unspecified etiology) compared to the control group. However, the level of freely circulating serotonin was the same in both groups [31]. In a recently published observation by A. Marwa Gamaleldin et al., serotonin levels were investigated in patients with cirrhosis of viral etiology (HCV) and signs of portal hypertension. Serotonin levels were studied using the ELISA method. The results revealed an increase in serotonin levels as liver cirrhosis and portal hypertension had been progressing [32]. Considering the results previously obtained by Lang P. A. et al. [28], which were based on a mouse model, it can be assumed that the etiology of liver cirrhosis significantly influences serotonin levels in patients’ blood.

We assume that the low serotonin levels we observed in the group of patients with cirrhosis are due to a number of pathological processes. First of all, as the disease progresses to cirrhosis, there is a change in the composition and metabolic activity of the intestinal microbiota, including with respect to the synthesis of short-chain fatty acids. This leads to a reduction in tryptophan metabolism in the human intestine. Additionally, the described phenomena of defenestration and capillarization of liver sinusoidal cells, activation of hepatic stellate cells, and secretion of pro-inflammatory molecules contribute to the disruption of hepatocyte homeostasis, including bilirubin metabolism disorders. The progression of liver fibrosis is closely related to the development of portal hypertension syndrome, ascites, splenomegaly with hypersplenism, and reduced platelet levels. Thrombocytopenia, combined with reduced serotonin levels and impaired synthetic liver function in relation to the production of coagulation factors, leads to the hypocoagulation phenomenon. Taking into account the previously obtained data, we assume a significant influence of the etiological factor on the level of serotonin during cirrhosis of the liver.

### 4.2. Kynurenine

In many tissues and organs, the kynurenine pathway is the primary route of tryptophan catabolism. According to the literature, most kynurenine is metabolized in the liver. However, it has been established that pro-inflammatory cytokines, IFN-γ, and TNF-α, contribute to the increased expression of the enzyme indoleamine 2,3-dioxygenase [33]. It has been found that through metabolites of microbial origin, as well as by providing pathogen-associated molecular patterns such as lipopolysaccharides, the gut microbiota can indirectly affect hepatic sinusoidal endothelial cells and hepatocytes. Under certain stimulations, hepatocytes can secrete acute-phase proteins, complement proteins, and other pro-inflammatory molecules and express major histocompatibility complex MHC I/II [34]. These changes subsequently lead to the disruption of hepatocyte homeostasis, the activation of inflammation, and the progression of liver fibrosis [35,36]. We found that as liver fibrosis progresses, there is a significant increase in the level of kynurenine. High levels of kynurenine correlated with indicators of hypocoagulation. An increase in the level of xanthurenic acid was found in patients with hepatic encephalopathy. Hepatic encephalopathy is one of the most common complications of cirrhosis, of which one of the main factors in its development is an elevated level of ammonia. However, high ammonia levels are not found in all patients with cirrhosis. The role of other metabolites in the development of this complication is assumed [37,38].

According to the literature, xanthurenic acid is found in the cell membranes of eukaryotes and is also a metabolite of tryptophan catabolism. In a mouse model, it was shown that xanthurenic acid is able to pass through the blood–brain barrier [39]. Wang Q et al. showed that in patients with cirrhosis (of various etiologies) and hepatic encephalopathy, there was an increase in the levels of 3-hydroxykynurenine, 5-hydroxytryptophol, 5-methoxy-3-indoleacetic acid, indole lactic acid, kynurenine, and melatonin and a decrease in serotonin levels [40].

Chojnacki C. et al. studied the levels of serotonin pathway metabolites in patients with cirrhosis (ALD). The level of metabolites was studied using enzyme immunoassay. As the disease progressed and hepatic encephalopathy appeared, there was a decrease in serotonin levels and an increase in melatonin levels. The authors suggest that the prolonged persistence of elevated melatonin levels does not provide a neuroprotective function and, on the contrary, contributes to the development of sleep disorders. The authors also suggest a shift in tryptophan catabolism towards the formation of neurotoxic compounds of the kynurenine pathway as the disease progresses [38].

It has been described that patients with congenital hydroxykynureninuria, characterized by the excretion of large amounts of kynurenine, 3-hydroxykynurenine, and xanthurenic acid in the urine, primarily exhibit encephalopathy as the main manifestation of the disease [41].

We have noted that as the disease progresses in patients with liver cirrhosis and hepatic encephalopathy, the level of the kynurenine pathway metabolite, xanthurenic acid, increases. Currently, the effects of this metabolite on the neuropsychic activity of humans are not fully described. We assume that the shift in tryptophan metabolism towards increased production of kynurenine leads to changes in the ratio of kynurenine pathway derivatives and results in a shift in balance towards neurotoxic compounds, causing hepatic encephalopathy in patients with cirrhosis.

The results of our study demonstrate multidirectional changes in the levels of metabolites of the serotonin and kynurenine pathways. This may be due to the activation of pro-inflammatory links in pathogenesis and the activation of kynurenine pathway enzymes, leading to a shift in tryptophan metabolism towards the formation of kynurenine metabolites and a decrease in serotonin synthesis.

Thus, to the best of our knowledge, we are the first to determine diverse changes in the levels of serotonin and kynurenine pathway metabolites in patients with cirrhosis resulting from MAFLD and ALD, excluding patients with viral hepatitis and oncological neoplasms. The results of our study provide an information basis for the subsequent targeted analysis of these metabolites in the field of studying the metabolic profile of patients with liver diseases.

The limitation of our study lies in the small number of patients with decompensated cirrhosis resulting from MAFLD, as well as the fact that our patients did not follow a standardized diet. Another limitation of our study is the absence of data on mortality outcomes and quality of life questionnaire data. Further studies on lipidomics in patients of these groups are also of interest.

## 5. Conclusions

Previously, studies using gas–liquid chromatography–mass spectrometry involving a sufficient sample size of patients with cirrhosis and without including patients with cirrhosis of viral etiology (HCV and HBV) have not been included in the literature. We confirmed previously discussed changes in the balance of metabolites, specifically the diverse changes in the levels of the kynurenine and serotonin pathway metabolites of tryptophan catabolism in patients with cirrhosis. Thus, we hypothesize that changes in the levels of the serotonin and kynurenine pathway metabolites of tryptophan metabolism can potentially expand our understanding of the pathogenesis of liver disease, its progression, and the development of complications associated with cirrhosis.

## Figures and Tables

**Table 1:** Analysis of laboratory tests for various types of liver damage (MAFLD and ALD).

	Control (n = 14)	Steatosis MAFLD (n = 22)	Steatohepatitis MAFLD (n = 13)	Cirrhosis MAFLD (n = 9)	Steatosis ALD (n = 2)	Hepatitis ALD (n = 7)	Cirrhosis ALD (n = 31)	*p*-Value
Age, years	40.50 [29.75–44.75]	57.50 ** [49.25–65.00]	48.00 [39.00–58.00]	59.00 [54.00–66.00]	40.00 [36.50–43.50]	56.00 [48.00–65.00]	52.00 ** [41.50–60.00]	<0.001
Male/Female	3/11	9/13	6/7	1/8	2/0	4/3	16/15	0.251
Body mass index, kg/m^2^	22.71 ± 2.99 [20.99–24.44]	30.59 ± 5.30 ** [28.17–33.00]	31.43 ± 7.67 ** [26.79–36.06]	32.56 ± 4.43 ** [29.16–35.97]	29.82 [28.21–31.42]	27.44 [24.28–34.36]	25.70 [22.02–31.63]	<0.001
Serum HDL cholesterol, mmol/L	1.47 [1.47–1.47]	1.10 ** [0.92–1.14]	1.14 [0.92–1.65]	1.25 [0.86–1.35]	1.15 [1.07–1.22]	1.47 [0.99–1.74]	1.05 ** [0.82–1.33]	0.004
Serum LDL cholesterol, mmol/L	2.75 [2.75–2.75]	3.61 ** [3.38–4.38]	3.38 [2.89–3.41]	3.41 [3.31–4.29]	3.85 [3.74–3.96]	4.21 ** [3.96–4.27]	2.80 [2.10–4.21]	0.004
Serum VLDL cholesterol, mmol/L	0.36 [0.36–0.36]	0.85 * [0.67–1.19]	0.87 * [0.68–0.89]	0.94 * [0.87–1.18]	0.54 [0.45–0.64]	0.62 [0.53–0.80]	0.46 [0.41–0.54]	<0.001
Serum glucose, μmol/L	4.80 [4.35–5.48]	5.10 [4.81–5.70]	5.70 [5.50–7.18]	6.90 ** [5.36–7.50]	5.02 [4.96–5.07]	6.07 [5.39–6.45]	5.30 [4.99–6.08]	0.011
Alanine Aminotransferase, U/L	17.00 [11.00–21.00]	31.00 ■ [18.00–32.00]	76.00 * [50.00–84.00]	35.00 ** [28.00–47.00]	27.00 [20.50–33.50]	72.00 * [60.00–75.00]	23.00 ■■ [18.00–35.00]	<0.001
Aspartate aminotransferase, U/L	20.00 [17.00–23.00]	24.00 ■■ [21.00–26.00]	36.00 ** [30.00–51.00]	44.00 ** [40.00–51.00]	20.00 [16.50–23.50]	79.00 * [48.00–90.50]	55.00 * [34.50–61.50]	<0.001
IgG, g/L	11.10 [11.10–11.10]	11.10 [10.27–11.60]	11.10 [9.50–11.20]	12.60 [11.10–15.80]	10.69 [10.48–10.89]	14.60 [14.28–15.05]	16.10 * [14.38–19.16]	<0.001
IgM, g/L	1.40 [1.40–1.40]	0.90 [0.72–1.25]	0.87 ** [0.71–1.21]	1.27 [0.90–1.68]	1.43 [1.42–1.44]	1.30 [1.29–1.76]	2.10 [1.33–3.16]	<0.001
IgA, g/L	2.00 [2.00–2.00]	2.50 [2.26–2.57]	2.50 [2.27–2.52]	2.50 [2.50–3.72]	2.00 [2.00–2.00]	3.70 ** [3.70–3.72]	5.70 * □ [4.46–7.81]	<0.001
Platelets, 10^9^/L	307.50 [253.25–369.50]	247.50 [216.50–265.75]	256.00 [226.00–272.00]	162.00 * □ [113.00–178.00]	265.50 [265.25–265.75]	277.00 [255.50–303.50]	139.00 * ■■ [81.00–195.00]	<0.001
Red blood cells, 10^9^/L	4.54 [4.33–4.79]	4.80 [4.56–5.05]	4.67 [4.46–4.98]	4.51 [4.22–4.69]	5.24 [4.97–5.51]	5.97 [5.64–8.28]	4.99 ** □ ■■ [3.96–6.87]	<0.001
INR	1.06 ± 0.10 [1.00–1.12]	1.01 ± 0.07 [0.98–1.05]	1.02 ± 0.08 [0.97–1.07]	1.13 ± 0.09 [1.06–1.20]	1.08 [1.07–1.08]	1.00 [0.93–1.06]	1.36 * ■ [1.22–1.65]	<0.001

Note: Statistically significant comparisons within the group of patients with a single etiological factor of liver damage are indicated: * *p* < 0.001, ** *p* < 0.05 vs. control. □ *p* < 0.05 vs. steatosis. ■ *p* < 0.001, ■■ *p* < 0.05 vs. hepatitis.

**Table 2:** Tryptophan metabolites analysis depending on the etiology of liver disease.

	Control (n = 14)	MAFLD (n = 44)	ALD (n = 40)	MAFLD vs. ALD	MAFLD vs. CON	ALD vs. CON
3-OH Anthranilic acid	0.03 [0.02–0.03]	0.06 [0.04–0.12]	0.04 [0.02–0.07]	0.047 *	0.002 *	0.066
5-Hydroxytryptophan	3.25 [2.96–3,68]	3.87 [3.06–5.07]	3.33 [2.14–4.18]	0.036 *	0.161	0.970
5-Methoxytryptamine	0.80 [0.64–0.92]	0.82 [0.68–0.91]	0.64 [0.52–0.75]	<0.001 *	0.806	0.044 *
Kynurenine	1.40 [1.10–1.60]	1.70 [1.31–2.11]	1.94 [1.44–2.49]	0.402	0.038 *	0.011 *
Serotonin	1.80 [1.16–2.25]	1.08 [0.79–1.62]	0.45 [0.24–0.89]	<0.001 *	0.049 *	<0.001 *

Note: *—differences in indicators are statistically significant (*p* < 0.05).

**Table 3:** Tryptophan metabolite correlation analysis depending on the variant of liver damage of various etiologies.

	Control (n = 14)	Steatosis MAFLD (n = 22)	Steatohepatitis MAFLD (n = 13)	Cirrhosis MAFLD (n = 9)	Steatosis ALD (n = 2)	Hepatitis ALD (n = 7)	Cirrhosis ALD (n = 31)	*p*-Value
Kynurenine	1.40 [1.10–1.60]	1.66 [1.08–2.02]	1.49 [1.29–1.95]	2.02 ** [1.83–2.61]	1.61 [1.53–1.70]	1.41 [0.93–1.70]	2.26 ** [1.59–2.80]	0.003
Serotonin	1.80 [1.16–2.25]	1.08 [0.84–1.47]	1.73 [1.12–2.10]	0.73 ** [0.28–1.01]	2.32 [2.21–2.44]	1.22 [0.86–1.60]	0.34 * □ ■ [0.21–0.58]	<0.001

Note: Statistically significant comparisons within the group of patients with a single etiological factor of liver damage are indicated. * *p* < 0.001, ** *p* < 0.05 vs. control. □ *p* < 0.05 vs. steatosis. ■ *p* < 0.05 vs. hepatitis.

## Data Availability

Data are available upon reasonable request from the corresponding author. The data are not publicly available because it is not recommended by the local ethics committee.

## Supplementary Materials

The following supporting information can be downloaded at: https://www.mdpi.com/article/10.3390/biom14111449/s1, Supplementary Table S1: Laboratory tests analysis for various types of liver damage (MAFLD and ALD); Supplementary Table S2: Tryptophan metabolites’ correlation analysis depending on laboratory test for MAFLD; Supplementary Table S3: Tryptophan metabolites’ correlation analysis depending on laboratory test for ALD.
