# Supplementary material for: Tryptophan Metabolites in the Progression of Liver Diseases
Source: Biomolecules. 2024 Nov 15;14(11):1449. doi: 10.3390/biom14111449 (PMC11591776; doi:10.3390/biom14111449)
Supplement: Supplementary file 1 [file biomolecules-14-01449-s001.zip › biomolecules-3234562-supplementary.pdf]

Supplementary Materials:

Table S1. Laboratory tests analysis for various types of liver damage (MAFLD and ALD).

	**Control (n=14)**	**Steatosis MAFLD (n=22)**	**Steatohepatitis MAFLD (n=13)**	**Cirrhosis MAFLD (n=9)**	**Steatosis ALD (n=2)**	**Hepatitis ALD (n=7)**	**Cirrhosis ALD (n=31)**	**p-value**
Age, years	40.50 [29.75 – 44.75]	57.50 ** [49.25 – 65.00]	48.00 [39.00 – 58.00]	59.00 [54.00 – 66.00]	40.00 [36.50 – 43.50]	56.00 [48.00 – 65.00]	52.00 ** [41.50 – 60.00]	< 0.001
Male/Female	3/11	9/13	6/7	1/8	2/0	4/3	16/15	0.251
Body mass index, kg/m²	22.71 ± 2.99 [20.99 – 24.44]	30.59 ± 5.30 ** [28.17 – 33.00]	31.43 ± 7,67 ** [26.79 – 36.06]	32.56 ± 4.43 ** [29.16 – 35.97]	29.82 [28.21 – 31.42]	27.44 [24.28 – 34.36]	25.70 [22.02 – 31.63]	< 0.001
Serum cholesterol, mmol/L	4.61 [4.22 – 5.30]	5.80 [5.19 – 6.58]	4.98 [4.68 – 5.65]	5.25 [4.37 – 6.05]	5.41 [5.25 – 5.57]	5.21 [4.77 – 6.87]	4.45 [3.81 – 5.46]	0.041
Serum HDL cholesterol, mmol/L	1.47 [1.47 – 1.47]	1.10 ** [0.92 – 1.14]	1.14 [0.92 – 1.65]	1.25 [0.86 – 1.35]	1.15 [1.07 – 1.22]	1.47 [0.99 – 1,74]	1.05 ** [0.82 – 1,33]	0.004
Serum LDL cholesterol, mmol/L	2.75 [2.75 – 2.75]	3.61 ** [3.38 – 4.38]	3.38 [2.89 – 3.41]	3.41 [3.31 – 4.29]	3.85 [3.74 – 3.96]	4.21 ** [3.96 – 4.27]	2.80 [2.10 – 4.21]	0.004
Serum VLDL cholesterol, mmol/L	0.36 [0.36 – 0.36]	0.85 * [0.67 – 1.19]	0.87 * [0.68 – 0.89]	0.94 * [0.87 – 1.18]	0.54 [0.45 – 0.64]	0.62 [0.53 – 0.80]	0.46 [0.41 – 0.54]	< 0.001
Serum glucose, mmol/L	4.80 [4.35 – 5.48]	5.10 [4.81 – 5.70]	5.70 [5.50 – 7.18]	6.90 [5.36 – 7.50] **	5.02 [4.96 – 5.07]	6.07 [5.39 – 6.45]	5.30 [4.99 – 6.08]	0.011
Serum total protein, g/L	73.00 [71.00 – 75.38]	72.00 [70.00 – 76.00]	70.00 [69.30 – 74.00]	74.00 [68.00 – 75.00]	73.50 [73.25 – 73.75]	74.00 [72.00 – 77.70]	71.00 [66.65 – 75.00]	0.523
Serum albumin, g/L	44.90 [43.47 – 46.62]	45.00 [43.60 – 46.00]	44.90 [44.00 – 46.80]	44.40 [40.00 – 45.00]	46.05 [45.83 – 46.27]	45.50 [40.85 – 47.00]	35.00 * [33.95 – 38.65]	< 0.001

Serum total bilirubin, μmol/l	10.40 [9.57 – 12.65]	12.40 [10.30 – 15.10]	12.20 [8.00 – 20.10]	15.60 [11.90 – 18.90]	23.80 [17.85 – 29.75]	20.70 [15.15 – 21.65]	35.30 * [23.50 – 74.60]	< 0.001
Serum direct bilirubin, μmol/l	2.50 [2.25 – 2.75]	1.90 [1.60 – 2.50]	2.40 [1.80 – 3.30]	3.30 [2.40 – 3.40]	3.50 [2.80 – 4.20]	3.40 [2.60 – 5.60]	11.20 * [6.35 – 28.75]	< 0.001
Serum iron, μmol/l	11.60 [10.07 – 18.50]	17.85 [13.83 – 19.95]	16.60 [13.10 – 17.40]	18.10 [15.35 – 22.90]	20.00 [18.00 – 22.00]	20.90 [17.85 – 23.61]	19.30 [13.30 – 24.85]	0.157
Alanine aminotransferase, U/L	17.00 [11.00 – 21.00]	31.00 ■ [18.00 – 32.00]	76.00 * [50.00 – 84.00]	35.00 ** [28.00 – 47.00]	27.00 [20.50 – 33.50]	72.00 * [60.00 – 75.00]	23.00 ■■ [18.00 – 35.00]	< 0.001
Aspartate aminotransferase, U/L	20.00 [17.00 – 23.00]	24.00 ■■ [21.00 – 26.00]	36.00 ** [30.00 – 51.00]	44.00 ** [40.00 – 51.00]	20.00 [16.50 – 23.50]	79.00 * [48.00 – 90.50]	55.00 * [34.50 – 61.50]	< 0.001
Gamma-glutamyl transferase, U/L	13.00 [13.00 – 14,75]	27.00 [18.00 – 37.00]	41.00 [23.00 – 52.00]	66.00 ** [48.00 – 123.00]	21.00 [20.00 – 22.00]	154.00 * [41.50 – 401.00]	99.00 * [55.50 – 308.00]	< 0.001
Alkaline phosphatase, U/L	60.00 [51.00 – 63.00]	85.00 ** [69.00 – 138.00]	103.00 [78.00 – 187.00]	122.00 [73.00 – 169.00]	119.00 [108.00 – 130.00]	68.00 [62.00 – 159.50]	140.00 * [105.00 – 287.00]	< 0.001
Creatinine, μmol/l	81.50 [74.50 – 89.25]	87.50 [77.00 – 96.58]	83.00 [77.00 – 93.00]	76.00 [69.00 – 86.00]	97.50 [97.25 – 97.75]	82.00 [70.00 – 108.00]	75.00 [65.00 – 82.00]	0.066
IgG, g/L	11.10 [11.10 – 11.10]	11.10 [10.27 – 11.60]	11.10 [9.50 – 11.20]	12.60 [11.10 – 15.80]	10.69 [10.48 – 10.89]	14.60 [14.28 – 15.05]	16.10 * [14.38 – 19.16]	< 0.001
IgM, g/L	1.40 [1.40 – 1.40]	0.90 [0.72 – 1.25]	0.87 ** [0.71 – 1.21]	1.27 [0.90 – 1.68]	1.43 [1.42 – 1.44]	1.30 [1.29 – 1.76]	2.10 [1.33 – 3.16]	< 0.001
IgA, g/L	2.00 [2.00 – 2.00]	2.50 [2.26 – 2.57]	2.50 [2.27 – 2.52]	2.50 [2.50 – 3.72]	2.00 [2.00 – 2.00]	3.70 ** [3.70 – 3.72]	5.70 * □□ [4.46 – 7.81]	< 0.001
Platelets, 10^9/L	307.50 [253.25 – 369.50]	247.50 [216.50 – 265.75]	256.00 [226.00 – 272.00]	162.00 * □□ [113.00 – 178.00]	265.50 [265.25 – 265.75]	277.00 [255.50 – 303.50]	139.00 * ■■ [81.00 – 195.00]	< 0.001
White blood cells, 10^9/L	6.01 [5.08 – 7.68]	6.13 [4.97 – 7.55]	5.62 [4.97 – 6.34]	5.91 [4.28 – 6.21]	5.24 [4.97 – 5.51]	5.97 [5.64 – 8.28]	4.99 [3.96 – 6.87]	0.285

Red blood cells, 10^9/L	4.54 [4.33 – 4.79]	4.80 [4.56 – 5.05]	4.67 [4.46 – 4.98]	4.51 [4.22 – 4.69]	5.24 [4.97 – 5.51]	5.97 [5.64 – 8.28]	4.99 ** □□ ■■ [3.96 – 6.87]	< 0.001
INR	1.06 ± 0.10 [1.00 – 1.12]	1.01 ± 0.07 [0.98 – 1.05]	1.02 ± 0.08 [0.97 – 1.07]	1.13 ± 0.09 [1.06 – 1.20]	1.08 [1.07 – 1.08]	1.00 [0.93 – 1.06]	1.36 * ■ [1.22 – 1.65]	< 0.001

Note: Statistically significant comparisons within the group of patients with a single etiological factor of liver damage are indicated
* p < 0.001, ** p < 0.05 vs. Control
□ p < 0.001, □□ p < 0.05 vs. Steatosis
■ p < 0.001, ■■ p < 0.05 vs. Hepatitis.

Table S2. Tryptophan metabolites' correlation analysis depending on laboratory test for MAFLD.

	**3-OH Anthranilic acid**	**5-Hydroxy-tryptophan**	**5-Methoxy-tryptamine**	**Kynurenine**	**Serotonin**	**Xanthurenic acid**
Age, years	0.974	0.963	0.858	0.361	0.709	0.587
Male/Female (16/28)	0.702	0.919	0.541	0.966	0.154	0.702
Body mass index, **kg/m^2**	NS	NS	$\varrho = 0.318$ $p = 0.035$	NS	NS	NS
Serum LDL cholesterol, **mmol/L**	$\varrho = 0.372$ $p = 0.014$	NS	NS	NS	NS	NS
Serum VLDL cholesterol, **mmol/L**	$\varrho = 0.356$ $p = 0.018$	$\varrho = 0.473$ $p = 0.001$	NS	NS	NS	NS
Serum total bilirubin, **µmol/l**	NS	NS	NS	$\varrho = 0.440$ $p = 0.003$	NS	NS
Serum direct bilirubin, **µmol/l**	NS	NS	NS	$\varrho = 0.386$ $p = 0.010$	NS	NS
Serum iron, **µmol/l**	NS	$\varrho = 0.327$ $p = 0.030$	NS	NS	NS	NS
Gamma-glutamyl	NS	NS	NS	$\varrho = 0.313$ $p = 0.039$	NS	NS

transferase, **U/L**						
IgM, **g/L**	NS	NS	NS	ϱ = 0.395 p = 0.008	NS	ϱ = 0.323 p = 0.033
IgA, **g/L**	NS	NS	NS	ϱ = 0.375 p = 0.012	NS	NS
Platelets, **10^9/L**	NS	NS	NS	NS	ϱ = 0.348 p = 0.022	NS
Red blood cells, 10^9/L	NS	NS	NS	NS	ϱ = 0.341 p = 0.025	NS
INR	NS	NS	NS	ϱ = 0.338 p = 0.025	NS	NS

Note: NS—non-significant

Table S3. Tryptophan metabolites' correlation analysis depending on laboratory test for ALD.

	**3-OH Anthranilic acid**	**5-Hydroxy-tryptophan**	**5-Methoxy-tryptamine**	**Kynurenine**	**Serotonin**	**Xanthurenic acid**
Age, years	0.974	0.963	0.858	0.361	0.709	0.587
Male/Female (22/18)	0.702	0.919	0.541	0.966	0.154	0.702
Serum HDL cholesterol, mmol/L	NS	NS	NS	NS	ϱ = 0.329 p =0.038	NS
Serum LDL cholesterol, mmol/L	NS	NS	ϱ = 0.341 p = 0.031	NS	NS	NS
Serum total protein, g/L	NS	NS	NS	NS	ϱ = 0.463 p = 0.003	NS
Serum albumin, g/L	NS	NS	r_{xy} = 0.354 p = 0.025	NS	ϱ = 0.548 p = < 0.001	ϱ = 0.465 p = 0.002
Serum total bilirubin, μmol/l	NS	NS	ϱ = -0.431 p = 0.005	NS	ϱ = -0.546 p = < 0.001	ϱ = -0.339 p = 0.032
Serum direct bilirubin, μmol/l	NS	ϱ = -0.320 p = 0.044	ϱ = -0.328 p = 0.039	ϱ = 0.331 p = 0.037	ϱ = -0.664 p = <0.001	NS
Alanine aminotransferase, U/L	NS	NS	NS	NS	ϱ = 0.361 p = 0.022	NS
Creatinine, μmol/l	NS	NS	NS	NS	ϱ = 0.339 p = 0.032	NS

IgG, g/L	NS	NS	NS	NS	ϱ = -0.427 p = 0.006	NS
IgM, g/L	NS	NS	NS	ϱ = 0.316 p = 0.047	ϱ = -0.352 p = 0.026	NS
IgA, g/L	NS	NS	ϱ = -0.328 p = 0.039	NS	ϱ = -0.411 p = 0.008	NS
Platelets, 10^9/L	NS	NS	ϱ = 0.349 p = < 0.001	ϱ = -0.255 p = 0.011	ϱ = 0.519 p = < 0.001	NS
Red blood cells, 10^9/L	NS	NS	ϱ = 0.328 p = 0.039	ϱ = -0.386 p = 0.014	ϱ = 0.741 p = <0.001	ϱ = 0.345 p = 0.029
INR	NS	NS	ϱ = -0.520 p = < 0.001	ϱ = 0.340 p = 0.032	ϱ = -0.743 p = <0.001	ϱ = -0.319 p = 0.045

Note: NS—non-significant
